# Mesenchymal Stem Cell-Secreted TGF-*β*1 Restores Treg/Th17 Skewing Induced by Lipopolysaccharide and Hypoxia Challenge via miR-155 Suppression

**DOI:** 10.1155/2022/5522828

**Published:** 2022-03-12

**Authors:** Ming Xue, Xiwen Zhang, Jianxiao Chen, Feng Liu, Jingyuan Xu, Jianfeng Xie, Yi Yang, Weiping Yu, Haibo Qiu

**Affiliations:** ^1^Department of Critical Care Medicine, Zhongda Hospital, School of Medicine, Southeast University, Nanjing 210009, China; ^2^Department of Pathophysiology, School of Medicine, Southeast University, Nanjing 210009, China

## Abstract

**Background:**

Regulatory T cell (Treg)/T helper (Th) 17 skewing is important in the development of acute respiratory distress syndrome (ARDS). Immunomodulatory effects of mesenchymal stem cell- (MSC-) secreted transforming growth factor- (TGF-) *β*1 on CD4^+^ T cells are environment-sensitive and lack discussion in hypoxic and inflammatory conditions.

**Methods:**

Mouse splenic CD4^+^ T cells were precoated with anti-CD3 (5 *μ*g/ml) and anti-CD28 (2 *μ*g/ml) overnight. RAW264.7 cells were added as antigen-presenting cells (APCs). T cells with and without RAW264.7 cells were treated with various LPS concentrations of 0, 10, 100, and 1000 ng/ml or/and at hypoxia condition of 5% O_2_. Based on LPS (100 ng/ml) and hypoxia conditions (5% O_2_) as stimuli, MSCs were set as direct coculture or indirect coculture by transwell system. Anti-TGF-*β*1 neutralization antibody was added to explore the role of TGF-*β*1 among the soluble factors secreted by MSCs; miR-155 overexpression of CD4^+^ T cells was performed by transfection, and then, cells were added to the MSC-CD4^+^ T cell coculture system in hypoxic- and LPS-stimulated condition. After 48 hours, cells or supernatants were collected for detection of frequency of Treg and Th17 subsets, CD4^+^ T cell apoptosis and proliferation capacity assay by flow cytometry, secretion of INF-*γ*, IL-17A, IL-21, TGF-*β*1, and IL-10 by ELISA, and levels of miR-155, Rorc, Foxp3, and Ptpn2 mRNA expression of CD4^+^ T cells by RT-PCR.

**Results:**

MSCs could restore skewed Treg/Th17 induced by LPS and hypoxia compared to groups without MSCs with increased secretion of TGF-*β*1, IL-10, and IL-17A (*P* < 0.05) and attenuate the increased expression of miR-155 in CD4^+^ T cells via cell-to-cell contact mechanism while TGF-*β*1 neutralization significantly inhibited the effects of MSCs restoring skewed Treg/Th17 and abolished its effect on miR-155 expression in CD4^+^ T cells.

**Conclusions:**

These findings suggested miR-155 suppression of CD4^+^ T cells mediated MSC-secreted TGF-*β*1 modulating skewed Treg/Th17 induced by LPS-hypoxia challenge, providing evidence when proposing future T lymphocyte-targeted cell therapy in a specific condition.

## 1. Background

Acute respiratory distress syndrome and sepsis are life-threatening clinical syndromes and continue to be a significant burden on society with high morbidity and mortality [1, 2]. Progressive hypoxemia and uncontrolled inflammation caused by heterogeneous etiologies interacted and composed the pathological environment [1, 3]. Upon injury, local stem and progenitor cell populations of lung epithelial cells would be initiated to respond to the loss or damage of epithelial or other injured tissues. Recent reports have shown that lineage-negative epithelial lacking expression of genes coding secretoglobin family 1A member 1 (SCGB1A1) or surfactant protein C (SFTPC) are resident in the distal lung and are capable of migration to sites of experimental injury and differentiated into alveolar epithelium while these populations are rare [4]. Exogenous MSC treatment has shown benefit on survival and organ protection in preclinical ARDS animal models and has been applied clinically among ARDS patients [5]. Cell-based therapy for ARDS requires a detailed understanding of injured cells in response to MSCs, taking into account the influence of the hypoxic and inflammatory environment during ARDS. Studies have demonstrated roles of MSCs in repairing endothelium and epithelium while the mechanism involved in modulating immune cells remains to be further investigated in specific pathological condition [6].

An imbalance between regulatory T cells (Treg) and interleukin- (IL-) 17-producing T helper cells (Th17) is reported to be characteristic for ARDS development [7, 8]. Th17 cells are a population of CD4^+^ T cells that express the cytokine IL-17, which drives leukocyte recruitment and activation to bridge innate and adaptive immunity and is in charge of the clearance of extracellular pathogens. Excessive generation and activation of Th17 cells attribute to hyperinflammatory damage [9]. Recent clinical studies among ARDS patients have identified a significantly closed link between increased Th17 frequency and greater illness severity and showed the levels of IL-17A were increased in bronchoalveolar lavage [10]. Accordingly, Tregs, characterized by forkhead box P3 (Foxp3), contribute to the resolution of the hyperinflammatory responses by regulating responses mediated by T helper cells. MSCs have been shown regulating Treg/Th17 balance in Th17 dominance multiple disease models such as systemic lupus erythematosus and ARDS [11, 12]. However, Treg/Th17 skewing upon the hypoxic and inflammatory environment in vitro along with effects of MSCs remains not completely discussed.

MSC-mediated immunoregulation is primarily via both cell-cell contact and paracrine activity by the release of soluble factors under specific conditions [13]. MSCs secrete a small amount of TGF-*β*1 under normal conditions. High TGF-*β*1 levels have been detected in MSC medium under inflammatory or hypoxic conditions [14–16]. Meanwhile, TGF-*β*1 is a critical and initiative factor for both Th17 and Treg development by inducing the expression of the transcription factors retinoic-acid-receptor-related orphan receptor *γ*t (ROR*γ*t) and Foxp3, respectively [17, 18]. In an ARDS mouse model, MSCs overexpressing TGF-*β*1 could regulate lung inflammation and attenuate lung injuries by modulating the imbalance of Th17/Treg in the lungs [12]. Thus, MSC-generated TGF-*β*1 could theoretically restore skewed Treg/Th17 in inflammatory or hypoxia condition.

MicroRNAs (miRs) are conserved, single-stranded noncoding RNAs, play a role in cellular differentiation, and regulate the immune system by binding the 3′ untranslated region (UTR) of mRNA, leading to the suppression of the target genes. miR-155 and miR-146a were the most commonly reported to be increased in activated immune cells, including T cells, especially in the process of naïve CD4^+^ T cells developing to T helper cells and regulatory T cells [19, 20]. The role of miR-155 in T cells varied depending on distinct environments. Some studies demonstrated miR-155 is necessary for Treg function and expansion in graft-versus-host disease and induces specific Treg subpopulations expressing CD39, indicating immunosuppression occurrence in septic patients [21–24]. However, under some inflammatory circumstances, miR-155 expression is shown negatively associated with Treg induction regulated by TGF-*β*1 and promotes Th17 cell differentiation [21, 25]. Mesenchymal stem cell soluble factors could modulate some specific miR expression, like those of miR-155 and miR-23b in dendritic cells [23]. Whether miRs are involved in the underlying mechanisms of CD4^+^ T cells differentiating to Treg and Th17 subsets in response to MSCs upon hypoxic and inflammatory conditions needs further investigation.

Therefore, in this study, lipopolysaccharide (LPS) with/without hypoxia was used as a stimulus in vitro. The aim of the present study is to evaluate the generation of CD4^+^ T cells from Treg and Th17 upon LPS and/or hypoxia challenge in response to MSCs and discuss the potential mechanism.

## 2. Methods

### 2.1. Animals

C57BL/6 male mice, 4-6 weeks of age, were purchased from the Comparative Medicine Centre, Yangzhou University (Yangzhou, China). The animals were housed 5 mice per cage in a laminar air flow room maintained at 22 ± 2°C with a relative humidity of 55 ± 5%. Mice were cared and treated in accordance with the guidelines established by the Committee of Animal Care and Use of Southeast University. The Committee of Animal Care and Use of Southeast University approved this study.

### 2.2. Isolation, Purification, and Identification of Mouse Spleen-Derived CD4^+^ T Cells

CD4^+^ T cells derived from mouse spleen were isolated through positive CD4 selection, using CD4 (L3T4) microbeads, MS columns, and MiniMACS™ Separator, according to the manufacturer's instructions (Miltenyi Biotec, Bergisch Gladbach, Germany) [26]. The identification was evaluated by immunofluorescence against CD4 by flow cytometry (FCM) [26]. CD4^+^ T cells were isolated from mouse spleen and identified by FCM with a purity of 97.01% (Supplemental Figure 1).

### 2.3. Cell Culture with Lipopolysaccharide and Hypoxia Stimulation

CD4^+^ T cells isolated from mouse spleen, with the addition of RAW264.7 cells provided by Cells Resource Center of Shanghai Institutes for Biological Sciences, Chinese Academy of Science (Shanghai, China) [14] (10^6^/well) as antigen-presenting cells, were seeded in 24-well Petri dishes (4–5 × 10^6^) precoated with anti-CD3 (5 *μ*g/ml) and anti-CD28 (2 *μ*g/ml) in RPMI1640 (Hyclone, USA) with 10%FBS (Gibco, USA) and were incubated in 37°C in a humidified atmosphere of 5% CO_2_ for 24 hours. Then, lipopolysaccharide (LPS, *Escherichia coli* O111: B4, Sigma Aldrich, Munich, Germany) at the dose of 10, 100, and 1000 ng/ml with/without hypoxia (5% O_2_) was, respectively, added as stimuli. After 48 hours, cells were harvested for detection of Treg and Th17 frequency by flow cytometry.

### 2.4. Bone Marrow-Derived MSC-Treated CD4^+^ T Cell Coculture System

Mouse bone marrow MSCs were purchased from Cyagen Biosciences Inc. (Guangzhou, China), which has been identified by detecting cell surface markers with ≥90% positivity for expression of cell surface antigens CD29, CD44, and CD105 and ≤5% positivity for expression of cell surface antigens CD45 and CD34 [12], and cells were maintained as previously described [15]. MSCs (10^6^) were directly added to the prepared CD4^+^ T cells or planted in the upper chamber of 0.4 *μ*m pore size transwell (Millipore, USA) inserted to set up a direct or indirect coculture system and then received stimulation of LPS (100 ng/ml) combined with hypoxia (5% O_2_) at 37°C in a 5% CO_2_ incubator.

### 2.5. Reagent Treatment

#### 2.5.1. Anti-TGF-*β*1 Blocking by Neutralization Antibody

To evaluate the effect of TGF-*β*1 among MSC-secreted soluble factors, anti-TGF-*β*1 antibodies (ab64715, Abcam, United Kingdom) of 10 *μ*g/ml were added into the medium of indirect MSC-treated CD4^+^ T cell group before LPS (100 ng/ml) and hypoxia (5% O_2_) stimulation and then followed by Treg/Th17 and miR-155 expression evaluation.

In addition, ALK5 inhibitor was used for blocking the TGF-*β-*mediated signaling. In the case of ALK5 inhibitor treatment (SB431542, Sigma, Taufkirchen, Germany), cells were preincubated with 1 *μ*m SB-431542 dissolved in DMSO for 30 min on ice before putting the cells into the coculture system with MSCs receiving LPS (100 ng/ml) and hypoxia (5% O_2_) stimulation and then followed by Treg/Th17 detection.

### 2.6. Detection of Treg and Th17 Population by Flow Cytometry

Cells were harvested, washed extensively, and dissociated into a single cell suspension. For Th17, IL-17A without CD8 expression, lymphocytes were detected as described previously. Cells were stimulated for 4 hours with 2 *μ*g/ml leukocyte activation cocktail (BD Pharmingen™, USA) at 37°C and 5% CO_2_. Upon harvest, cells were surface stained with antibody anti-human CD8a-APC (BD Pharmingen™, USA) at room temperature in the dark and then fixed and permeabilized using intracellular fixation and permeabilization (BD Pharmingen™, USA). Following fixing and permeabilization, cells were incubated with PE-conjugated anti IL-17 (BD Pharmingen™, USA). For Treg, Foxp3-producing CD25 lymphocytes were detected as described previously. Cells were surface stained with antibody CD25-APC (BD Pharmingen™, USA) at room temperature in the dark and then fixed and permeabilized using intracellular fixation and permeabilization (BD Pharmingen™, USA). Following fixing and permeabilization, cells were incubated with PE-conjugated anti-Foxp3 (BD Pharmingen™, USA). Samples were run on a flow cytometer (ACEA NovoCyte, USA). Data were analysed using FlowJo software (FlowJo, USA) and NovoExpress software (ACEA Biosciences, USA).

### 2.7. Cytokine Quantification

TGF-*β*1, IL-10, IL-17A, INF-*γ*, and IL-21 in the supernatant were determined via an enzyme-linked immunosorbent assay (ELISA) by using commercially available ELISA sets (ExCellBio, Shanghai, China, and Elabscience, Wuhan, China) in accordance with the instructions of the manufacture. All samples were measured in duplicate.

### 2.8. Real-Time PCR Detection

Total RNA of cells was obtained by Trizol reagent and then followed by cDNA synthesis. Real-time PCR was performed with the ABI Prism 7000 Sequence Detection System (Life Technologies, Carlsbad, CA, USA) according to the manufacturer's instructions. The average threshold count (Ct) values of 2–3 technical replicates were used in all calculations. The average Ct value of the internal controls was used to calculate Ct values for the samples. Data analysis was performed using the 2^-*ΔΔ*Ct^ method. U6 RNA was used as an endogenous control for miR detection and *β*-actin for Foxp3, Rorc, and Ptpn2 mRNA detection. The primer sequences are as listed in the supplemental file.

### 2.9. Apoptosis and Proliferation Capacity Assay by Flow Cytometry

CD4^+^ T cells isolated from the lower chamber in groups of control mimic-CD4^+^ T cells, miR-155 mimic-CD4^+^ T cells, and miR-155 inhibitor-CD4^+^ T cells with MSC treatment were collected and stained with Annexin V/PI kit (BD Pharmingen™, USA) for apoptosis, respectively. CellTrace Violet™ cell proliferation kit (Invitrogen™, USA) was used for proliferation capacity assay with flow cytometry analysis after a 72-hour in vitro stimulation.

### 2.10. miR-155 Overexpression by miR-155 Mimic Transfection

To identify the role of miR-155 suppression CD4^+^ T cells with skewed Treg/Th17 differentiation upon LPS-hypoxia challenge in response to MSC treatment by transwell, miR-155 mimics with respective controls designed and synthesized by RiboBio Co., Ltd. (Guangzhou, China) were pretransfected, respectively, to purified CD4^+^ T cells with Lipofectamine 2000 reagent. The expression of miR-155 was tested by real-time polymerase chain reaction (RT-PCR) at 4 and 48 hours after transfection. The transfection efficiency of miR-155 overexpression in CD4^+^ T cells was confirmed (Supplemental Figure 2). The CD4^+^ T cells pretransfected with miR-155 mimic or control received MSCs cocultured by transwell with/without LPS (100 ng/ml)-hypoxia (5% O_2_) stimulation for 48 hours. The frequency of Treg and Th17 population as well as apoptosis and proliferation capacity of CD4^+^ T cells was determined by flow cytometry (FCM). The CD4^+^ T cells were sorted by CD4 (L3T4) microbeads, and gene expression of Ptpn2, Foxp3, and Rorc mRNA of CD4^+^ T cells was assayed by RT-PCR.

### 2.11. Statistical Analysis

Comparisons of variables between groups were performed using unpaired *t*-tests, Mann–Whitney *U* tests, or chi-square tests, as appropriate by GraphPad PRISM Version 5.3 (San Diego, CA, USA). Statistical significance was set at the level of *P* < 0.05.

## 3. Results

### 3.1. Lipopolysaccharide and Hypoxia Stimulation Induced Skewed Treg/Th17

**Figure 1:**
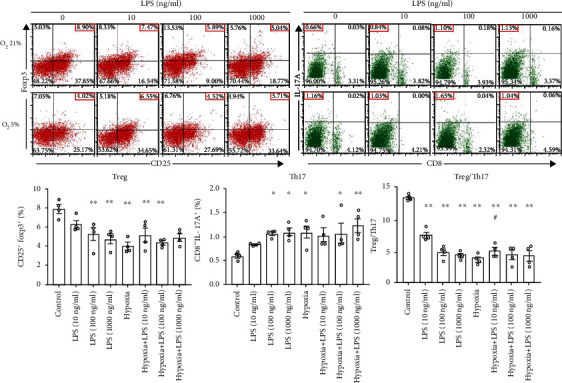
Effects of LPS at 10, 100, and 1000 ng/ml alone, hypoxia alone, or combined stimulation on mouse splenic CD4^+^ T cell differentiation to Treg or Th17 population. The phenotype of Treg and Th17 populations after 48-hour stimulation was assessed by flow cytometry. Treg cells were identified by CD25^+^Foxp3^+^ in CD4^+^ T cells and Th17 cells were by CD8^−^IL-17^+^ in CD4^+^ T cells. Representative flow plots were shown. Bar graphs represent mean frequency ± SEM of Treg and Th17 and mean values ± SEM of Treg/Th17 ratios in the various groups. ^∗^*P* < 0.05 vs. the control group, ^∗∗^*P* < 0.01 vs. the control group, and ^#^*P* < 0.05 vs. the LPS (10 ng/ml) group (*n* = 4). LPS: lipopolysaccharide; SEM: standard error of the mean; Th: T helper; Treg: regulatory T cells.

To figure out the impact of hypoxia, LPS, or combined stimulation on Treg and Th17 generation, CD4^+^ T cells with RAW264.7 cells added as antigen-presenting cells were cultured in vitro in the absence of or with LPS (10, 100, and 1000 ng/ml, respectively), hypoxia (5% O_2_), or combined stimulation for 48 hours. Compared to the control group with no stimulation, frequency of CD25^+^Foxp3^+^ in CD4^+^ T cells was decreased and that of CD8^−^IL17A^+^Th17 cells was increased markedly in CD4^+^ T cells upon LPS stimulation of 100 and 1000 ng/ml (*P* < 0.05, Figure 1). Hypoxia alone or combined with LPS challenge significantly reduced the frequency of CD25^+^Foxp3^+^ in CD4^+^ T cells in comparison with that of the control group (*P* < 0.05, Figure 1). Frequency of Th17 population in CD4^+^ T cells was markedly increased upon hypoxia alone or combined with LPS of 100 and 1000 ng/ml (*P* < 0.05, Figure 1). No difference in the percentages of Treg or Th17 cell populations was detected between the group stimulated with LPS at a certain dose alone and that in combination with hypoxia. Taken together, Treg/Th17 values pronouncedly decreased in groups with LPS alone, hypoxia alone, or combination stimulation compared to those in the group with no stimulation (*P* < 0.01, Figure 1). There the results showed LPS and (or) hypoxia stimulation in vitro could induce Treg/Th17 skewing, favouring Th17 rather than Treg populations. For both Treg decrease and Th17 increase that appeared when the concentration of LPS was above 100 ng/ml, LPS at the dose of 100 ng/ml and hypoxia was set as stimulus in the following experiments.

### 3.2. Mesenchymal Stem Cells Attenuated the Inflammation and Reserved Skewed Treg/Th17 upon LPS and Hypoxia Challenge In Vitro

**Figure 2:**
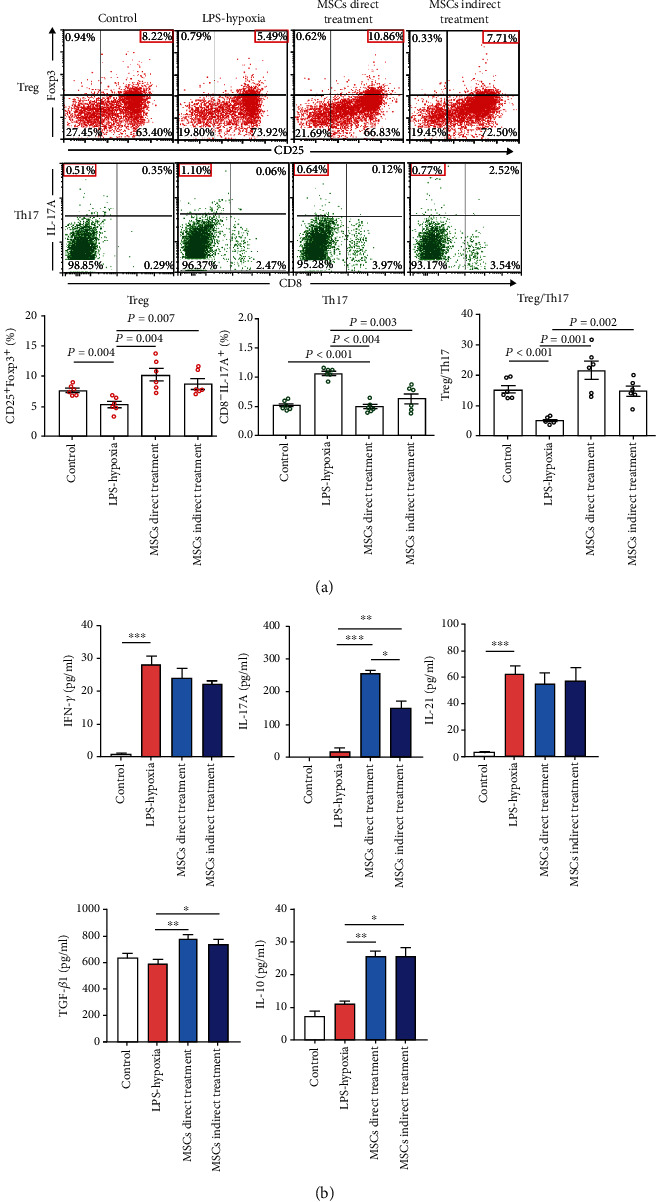
Effects of MSC on the skewed Treg/Th17 in LPS- and hypoxia-stimulated CD4^+^ T cells and cytokine secretion. CD4^+^ T cells alone (control), CD4^+^ T cells with LPS (100 ng/ml)-hypoxia (5% O_2_) stimulation (LPS-hypoxia), and MSC-treated CD4^+^ T cells with LPS-hypoxia challenge (MSC direct treatment and MSC indirect treatment) were cultured for 48 hours. (a) Then, cells were harvested for Treg and Th17 phenotypic identification by flow cytometry. Representative flow plots were shown. Bar graphs represent mean frequency ± SEM of Treg and Th17 and mean values ± SEM of Treg/Th17 ratios in the various groups. (b) The culture supernatants were collected for IFN-*γ*, IL-17A, IL-21, IL-10, and TGF-*β*1 level measurement by an enzyme-linked immunosorbent assay, presented with mean ± SEM in the bar graph. ^∗^*P* < 0.05 vs. the control group and ^∗∗^*P* < 0.01 vs. the control group. IFN: interferon; IL: interleukin; LPS: lipopolysaccharide; MSC: mesenchymal stem cell; SEM: standard error of the mean; TGF: transforming growth factor; Th: T helper; Treg: regulatory T cells.

To discuss the effect of MSCs on Treg/Th17 and the potential mechanisms in the condition of LPS-hypoxia combined stimulation, we introduced a transwell coculture system. CD4^+^ T cells with RAW264.7 cells were planted in the lower chamber, followed by LPS (100 ng/ml)-hypoxia (5% O_2_) stimulation. MSCs were, respectively, added to the upper and lower chambers for indirect and direct coculture treatment. We found that the MSC-treated CD4^+^ T cell groups, whether directly or indirectly compared to the LPS-hypoxia group, could markedly upregulate CD4^+^CD25^+^Foxp3^+^ Treg frequency and decrease that of CD8^−^IL-17A^+^Th17 in CD4^+^ T cells with no difference between indirect and direct addition of MSCs (*P* < 0.05, Figure 2(a)). These results indicated that MSCs restored Treg/Th17 imbalance induced by LPS and hypoxia challenge mainly via soluble factors rather than cell-to-cell contact. In addition, MSCs augmented the levels of anti-inflammatory cytokines, including TGF-*β*1 and IL-10 which were markedly increased in the supernatant of the group receiving MSC treatment when compared with that of the LPS-hypoxia group (*P* < 0.05, Figure 2(b)).

### 3.3. MSCs Restored Skewed Treg/Th17 Induced by LPS and Hypoxia via TGF-*β*1 Secretion

**Figure 3:**
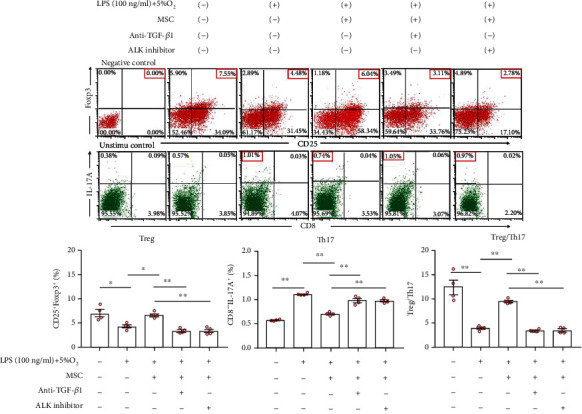
Effects of anti-TGF-*β*1 neutralization and blocking TGF-*β*1-mediated signal on LPS- and hypoxia-induced Treg/Th17 skewing in response to MSC indirect treatment. Representative flow plots were shown. Bar graphs represent mean frequency ± SEM of Treg and Th17 and mean values ± SEM of Treg/Th17 ratios in the various groups. LPS: lipopolysaccharide; SEM: standard error of the mean; TGF: transforming growth factor; Th: T helper; Treg: regulatory T cells. ^∗^*P* < 0.05 and ^∗∗^*P* < 0.01.

Among MSC-secreted soluble factors, TGF-*β*1 has been reported to be vital for Treg and Th17 differentiation in many disease models [11, 16]. In this study, soluble TGF-*β*1 levels were upregulated in parallel with the reduced Treg/Th17 by MSC treatment. To clarify the role of TGF-*β*1 among MSC-secreted factors on regulating Treg/Th17 in the context of in vitro ARDS environment, TGF-*β*1 secretion was blocked with TGF-*β*1 neutralization. Anti-TGF-*β*1 antibodies were added into the medium of the indirect MSC treatment group, followed by detection of Treg and Th17 by FCM. We found that TGF-*β*1 blocking by neutralization in MSC-treated CD4^+^ T cells or blocking TGF-*β*1-mediated signaling by ALK5 inhibitor caused a reversal effect of MSCs on restoring Treg/Th17 imbalance induced by LPS and hypoxia challenge (*P* < 0.05, Figure 3), suggesting that MSCs improved Treg/Th17 exposed to LPS and hypoxia stimulation by increased TGF-*β*1 secretion.

**Figure 4:**
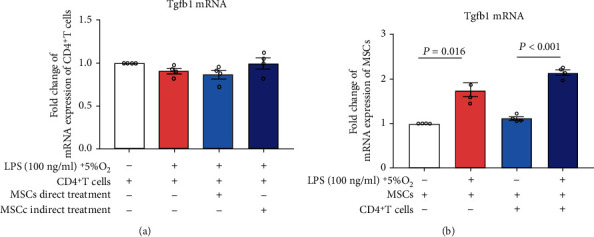
Effects of MSC-CD4^+^ T cell coculture and/or LPS-hypoxia stimulation on Tgfb1 mRNA expression of CD4^+^ T cells and MSCs. (a) Tgfb1 mRNA expression of CD4^+^ T cells receiving MSC treatments and/or LPS-hypoxia stimulation was detected by RT-PCR. (b) Tgfb1 mRNA expression of MSCs receiving indirect coculture with CD4^+^ T cells and/or LPS-hypoxia stimulation was detected by RT-PCR detection. LPS: lipopolysaccharide; MSC: mesenchymal stem cell; RT-RCR: real-time polymerase chain reaction; TGF: transforming growth factor.

To confirm whether the increased soluble TGF-*β*1 originated from MSCs or CD4^+^ T cells exposed to LPS and hypoxia stimulation, Tgfb1 mRNA expression of CD4^+^ T cells sorted from groups of control, ARDS, and MSCs treatment was measured by RT-PCR. Tgfb1mRNA levels of CD4^+^ T cells were comparable among the groups, indicating CD4^+^ T cells with LPS and hypoxia stimulation, with or without MSC treatment, would not be responsible for the increased TGF-*β*1 secretion. Moreover, Tgfb1 mRNA expressions of MSCs were explored with or without LPS (100 ng/ml) and hypoxia (5% O_2_) stimulation, with or without coculturing with CD4^+^ T cells by transwell system. When MSCs were cultured alone, Tgfb1mRNA expression of MSCs was markedly increased in the group with LPS and hypoxia stimulation than that without (*P* < 0.05, Figure 4(a)). When cocultured with CD4^+^ T cells in the transwell insert, the increased Tgfb1mRNA expression of MSCs was also detected in the group with LPS-hypoxia stimulation (*P* < 0.05, Figure 4(b)), compared to that without. These data confirmed the increased soluble TGF-*β*1 modulating skewed Treg/Th17 induced by LPS and hypoxia challenge was generated from MSCs.

### 3.4. MSC-Secreted TGF-*β*1 Inhibited miR-155 Expression of CD4^+^ T Cells upon LPS and Hypoxia Challenge

**Figure 5:**
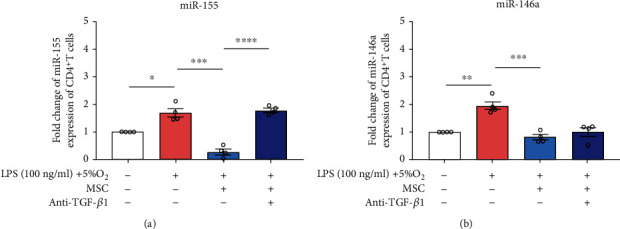
Effects of anti-TGF-*β*1 neutralization on miR-155 and miR-146a expression of CD4^+^ T cells upon LPS and hypoxia challenge in response to MSC treatment. Bar graphs represent mean ± SEM levels of (a) miR-155 and (b) miR-146a expression detected by RT-PCR. ^∗^*P* < 0.05, ^∗∗^*P* < 0.01, ^∗∗∗^*P* < 0.001, and ^∗∗∗∗^*P* < 0.0001 (*n* = 4). LPS: lipopolysaccharide; MSC: mesenchymal stem cell; RT-RCR: real-time polymerase chain reaction; SEM: standard error of the mean; TGF: transforming growth factor.

MSC soluble factors were proved to play immunomodulatory effects by regulating miR expression [27]. Here, we evaluated miR-155 and miR-146a expression, which has been reported to be associated with T cell phenotypic transformation of Treg/Th17 [20], in splenic CD4^+^ T cells with or without LPS (100 ng/ml)-hypoxia (5% O_2_) stimulation in vitro, with or without MSC coculture by transwell. The results showed miR-155 and miR-146a expression was significantly increased by in vitro ARDS environment established by LPS and hypoxia stimulation (*P* < 0.05, Figure 5). Indirect coculture with MSCs was able to reverse the increase of CD4^+^ T cell-expressed miR-155 and miR-146a induced by in vitro ARDS stimulation of LPS and hypoxia, while the effect of MSCs on miR-155 suppression was attenuated by cotreatment with TGF-*β*1 neutralization (*P* < 0.05, Figure 5). These results suggested that miR-155 expression of CD4^+^ T cells was suppressed in response to MSC-secreted TGF-*β*1 modulating Treg/Th17 differentiation under LPS-hypoxia-induced conditions.

### 3.5. miR-155 Mediated MSC Paracrine Effects on Treg/Th17 Skewing Induced by LPS-Hypoxia Challenge

**Figure 6:**
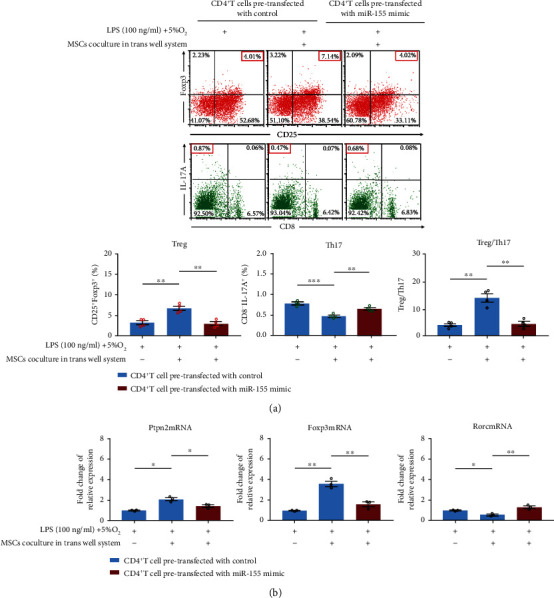
Effects of miR-155 overexpression in CD4^+^ T cells on LPS-hypoxia-induced Treg/Th17 skewing and related gene expression in response to MSC treatment. CD4^+^ T cells pretransfected with control/miR-155 mimic received MSC treatment by transwell system, with/without LPS-hypoxia challenge for 48 hours. (a) Then, cells were harvested for Treg and Th17 phenotypic identification by flow cytometry. Representative flow plots were shown. Bar graphs represent mean frequency ± SEM of Treg and Th17 and mean values ± SEM of Treg/Th17 ratios in the various groups. (b) The gene expression of Ptpn2, Foxp3, and Rorc mRNA of CD4^+^ T cells was detected by RT-PCR in the various groups. Bar graphs represent mean ± SEM levels of Ptpn2, Foxp3, and Rorc mRNA expression. Blue bar graphs present gene expression in CD4^+^ T cells pretransfected with control, and red bar graphs present gene expression in CD4^+^ T cells pretransfected with miR-155 mimic. ^∗^*P* < 0.05, ^∗∗^*P* < 0.01, and ^∗∗∗^*P* < 0.001 (*n* = 4). LPS: lipopolysaccharide; MSC: mesenchymal stem cell; RT-RCR: real-time polymerase chain reaction; SEM: standard error of the mean; Th: T helper; Treg: regulatory T cells.

We next examined whether miR-155 inhibition of CD4^+^ T cells was involved in MSCs regulating skewed Treg/Th17 induced by LPS-hypoxia challenge. miR-155 overexpression was performed by miR-155 mimic transfection. The CD4^+^ T cells pretransfected with miR-155 mimic and control were stimulated with LPS (100 ng/ml)-hypoxia (5% O_2_), followed by MSC coculture treatment in transwell for 48 hours. Flow cytometric analysis showed compared with CD4^+^ T cells pretransfected with controls with LPS-hypoxia stimulation, MSC coculture treatment could increase the frequency of CD4^+^CD25^+^Foxp3^+^Treg cells and reduced CD8^−^IL-17A^+^Th17 population while miR-155 overexpression by miR-155 mimic transfection reversed these changes (Figure 6(a)). Bioinformatic analysis indicated Ptpn2 mRNA might be one of the potential targets with 3′-UTR area binding miR-155 (Supplemental File: Figure S3), of which upregulation has been reported to attenuate proinflammatory response and promote Treg conversion [28]. We observed that, as compared to those of CD4^+^ T cells pretransfected with controls with LPS-hypoxia stimulation, MSC treatment could significantly enhance the expression of Foxp3 and Ptpn2 mRNA and reduce Rorc mRNA expression, which was in consistence with the Treg and Th17 phenotypic alteration, while miR-155 overexpression could inhibit the alterations of these genes' expression induced by MSCs (Figure 6(b)). These data further confirmed that miR-155 suppression in CD4^+^ T cells was necessary during MSC paracrine effect, modulating the skewed Treg/Th17 induced by LPS-hypoxia challenge.

### 3.6. Effects of miR-155 Expression on Proliferation Capacity and Survival of CD4^+^ T Cells

**Figure 7:**
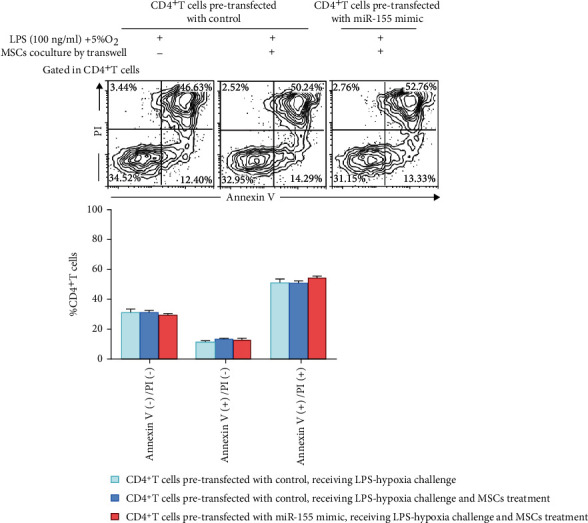
Effects of miR-155 overexpression in CD4^+^ T cells on apoptosis upon LPS-hypoxia challenge in response to MSC treatment. CD4^+^ T cells pretransfected with control/miR-155 mimic received MSC treatment by transwell system, with/without LPS-hypoxia challenge for 48 hours. Then, cells were harvested for apoptosis assay with Annexin V/PI staining by flow cytometry. Representative flow plots were shown. Bar graphs represent mean frequency ± SEM of living (Annexin V(-)/PI(-)), early apoptosis (Annexin V(+)/PI(-)), and late apoptosis and dead (Annexin V(+)/PI(-)) percentage of CD4^+^ T cells in the various groups. LPS: lipopolysaccharide; MSC: mesenchymal stem cell; SEM: standard error of the mean.

**Figure 8:**
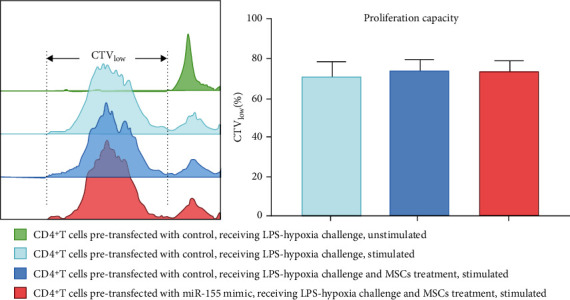
Effects of miR-155 overexpression in CD4^+^ T cells on proliferation capacity upon LPS-hypoxia challenge in response to MSC treatment. CD4^+^ T cells pretransfected with control/miR-155 mimic received MSC treatment by transwell system, with/without LPS-hypoxia challenge for 48 hours. Then, CD4^+^ T cells were sorted by positive CD4 selection, using CD4 microbeads and were labeled with CellTrace™ Violet (CTV) reagent prior to stimulation with anti-CD3/CD28 antibodies. Cells unstimulated were set as controls (green). Proliferation capacity of CD4^+^ T cells was identified as CTV_low_% by flow cytometry after 72-hour culture in vitro. Bar graphs represent mean frequency ± SEM of proliferation capacity of CD4^+^ T cells in the various groups. LPS: lipopolysaccharide; MSC: mesenchymal stem cell; SEM: standard error of the mean.

Apart from Treg and Th17 conversion, we further examined the effect of miR-155 overexpression on the proliferation and survival of CD4^+^ T cells with MSC treatment exposed to LPS-hypoxia condition. Annexin V/PI staining results presented no difference in living, early apoptosis, and late apoptosis and dead parts of CD4^+^ T cells among the groups (Figure 7). Cell proliferation was measured using CellTrace™ Violet, to trace multiple generations using dye dilution by flow cytometry. The percentage of CTV_low_ presented cell proliferative capacity. The CTV staining analysis showed miR-155 overexpression did no impact on the proliferation capacity of CD4^+^ T cells exposed to MSCs cocultured by transwell with/without LPS-hypoxia challenge (Figure 8).

## 4. Discussion

Despite the growing supportive evidence of MSCs in controlling inflammation and protecting organ function [4–6], uncovering its effects and mechanisms on different immune cells under the specific environment will benefit future cell-based therapy and the selected target populations. In this study, we investigated the generation of CD4^+^ T cells to Treg and Th17 upon LPS and hypoxia challenge in response to MSCs. We show that in vitro LPS, hypoxia, or their combined stimulation could induce skewed Treg/Th17, favouring Th17 cells rather than Treg cells. MSCs could restore skewed Treg/Th17 while secreting more TGF-*β*1 and inducing an inhibited effect on miR-155 expression in CD4^+^ T cells upon LPS and hypoxia challenge. The alterations of miR-155 expression in CD4^+^ T cells and Treg/Th17 could be diminished by anti-TGF-*β*1 or TGF-*β*1 receptor blockade. miR-155 overexpression could abolish MSC paracrine effects on skewed Treg/Th17 with reduction in Treg phenotypic gene expression of Ptpn2 mRNA and Foxp3 mRNA and upregulation of Th17 phenotypic gene expression of Rorc mRNA. Accordingly, we demonstrated that MSC-secreted TGF-*β*1 is capable of modulating skewed Treg/Th17 upon LPS and hypoxia challenge via miR-155 suppression of CD4^+^ T cells.

Several studies have verified that Treg/Th17 skewing plays a crucial role in sepsis and ARDS [7, 8, 10, 29, 30]. LPS, an endotoxin originating from the cell wall of gram-negative bacteria, was the most used for a bacterial hyperinflammatory model of experimental ARDS [31]. In this study, we utilized LPS and hypoxia stimulation and observed the induction of a phenotype skewing toward Th17, rather than Treg cells, which was consistent with the results observed in ARDS patients clinically characterized with inflammation and hypoxemia [7]. In line with skewed Treg/Th17 induced by LPS and hypoxia challenge, the level of proinflammatory cytokines, such as IFN-*γ*, IL-17A, and IL-21, was indeed increased. CD4^+^ T cells could be primed by the following factors in our experiment settings. First, with the presence of RAW264.7 macrophages, activation of the CD14/TLR4 receptor structure on these cells by LPS complex triggered the production of inflammatory mediators and then drove naive T cells converting into a different subset [32, 33]. In the presence of soluble IL-6 with/without TGF-*β*1 detected in the LPS-hypoxia stimulated group, IL-17A-expressing Th17 cells and FOXP3-expressing Treg cells could be, respectively, induced [34]. The IL-21 release participates in the differentiation/amplification of Th17 cells [35], of which the remarkable augmentation (Figure 2(b)) might contribute to the predominant role of Th17 in skewed Treg/Th17 upon LPS-hypoxia challenge. Second, LPS is reported to directly regulate Th17 differentiation in vitro in a TLR-4-specific manner, although LPS showing a modest effect on TLR4 expression was dose dependent with remarkable effects at a dose of higher than 10 *μ*g/ml [36]. In addition, hypoxia exposure could influence the processes of T cell differentiation and phenotypic stability [37]. Previous studies reported that HIF-1, as a cellular production in response to hypoxia, could boost proinflammatory IL-17-producing Th17 cells and suppress the Foxp3 expression on Treg cell subsets [38], in which the similar alteration of Treg/Th17 upon hypoxia exposure was observed in the current study. Our study showed that the combined ex vivo stimulation of LPS at 10, 100, and 1000 ng/ml and hypoxia (5% O_2_) could induce skewed Treg/Th17, without evident differences detected from those with LPS or hypoxia stimulation alone.

By establishing an in vitro coculture system with LPS-hypoxia stimulation on CD4^+^ T cells, we found that MSC coculture restored skewed Treg/Th17 and significantly augmented the anti-inflammatory cytokine secretion. We have proved that MSCs overexpressing TGF-*β*1 could regulate lung inflammation and attenuate lung injuries by modulating the imbalance of Th17/Treg in the lungs of ARDS mice [12]. Establishing an in vitro cell coculture model with LPS and hypoxia challenge would promote our knowledge in the interaction between Treg and Th17 cell skewing and MSCs in a specific condition. Upon LPS and hypoxia challenge, the MSC treatment group exhibited a higher frequency of Treg and lower Th17 percentage in comparison with those without. This phenotypic conversion of Treg and Th17 induced by MSCs was partly consistent with previous findings [11, 39] in several disease models, while Najar et al.'s (2019) study showed that adipose tissue-derived MSCs could promote the development of a proinflammatory Th17 phenotype from activated T cells. The discrepancy might be attributed to the distinct environment around MSC-T cells between different experiment settings, including cell ratio and environmental cytokine release, which needs to be carefully addressed when proposing future cell-based therapies. In keeping with Foxp3-expressing Treg phenotype prevailing induced by MSCs in the coculture systems stimulated by LPS and hypoxia, the levels of anti-inflammatory cytokines, such as IL-10 and TGF-*β*1, were remarkably increased.

MSCs exert an immunomodulatory effect mainly via cell contact or paracrine mechanism [6]. In our study, MSCs regulating the skewed Treg/Th17 upon LPS and hypoxia challenge were dependent on cell-to-cell contact, suggesting that paracrine is the primary mechanism during this process. Among the soluble factors released by MSCs, TGF-*β*1 is proved to mediate a therapeutic immunosuppressive effect, switching CD4^+^ T cells from a proinflammatory phenotype of Th17 to an inflammation-resolving phenotype of Tregs [16] while the secretion of TGF-*β*1 by MSC is environment-sensitive [40]. The current literature supported more TGF-*β*1 production by MSCs under hypoxic- or LPS-stimulated condition [13, 14]. In the present setting, more TGF-*β*1 secretion was detected in parallel with reversed Treg/Th17 in MSC-treated CD4^+^ T cells with LPS and hypoxia stimulation. Meanwhile, more TGF-*β*1 release from MSCs, rather than CD4^+^ T cells upon LPS and hypoxia challenge, in the presence or absence of CD4^+^ T cells was confirmed at the mRNA and protein levels by RT-PCR and ELISA. Furthermore, MSC paracrine effects on restoring the skewed Treg/Th17 induced by LPS and hypoxia challenge could be restrained by anti-TGF-*β*1 blocking. Based on these observations, we proved that TGF-*β*1 secreted by MSCs could be responsible for regulating the Treg/Th17 imbalance induced by LPS and hypoxia challenge.

One novel finding of our study was that miR-155 suppression in CD4^+^ T cells mediated the regulation of MSC-secreted TGF-*β*1 on skewed Treg/Th17 induced by LPS-hypoxia challenge, which was associated with upregulation of Ptpn2 mRNA and Foxp3 mRNA expression and inhibition of Rorc mRNA. In mouse models infected by LPS or staphylococcal enterotoxin B, miR-155 expression was detected with a significant increase in inflammatory cytokine production, indicating its association with the proinflammatory response [41, 42]. In addition, miR-155 has been reported to be involved in T cell differentiation, survival, and proliferation capacity [25, 43] and could be regulated by TGF-*β*1 [20]. In case of thymocyte development, miR-155 facilitates thymic Treg cell development in both T cell intrinsic and extrinsic manners [44]. Within our results, LPS and hypoxia stimulation could markedly upregulate miR-155 expression in CD4^+^ T cells. In addition to Treg/Th17 skewing, MSC-secreted TGF-*β*1 could induce an inhibited effect on miR-155 expression in CD4^+^ T cells stimulated with LPS and hypoxia while miR-155 overexpression in CD4^+^ T cells could relieve the regulation of MSC-secreted TGF-*β*1 on Treg/Th17 skewing and showed no impact on cell survival and proliferation capacity. This indicated that miR-155 suppression in CD4^+^ T cells was necessary during MSC-secreted TGF-*β*1 regulating skewed Treg/Th17 upon LPS and hypoxia challenge. Diverse microenvironments along with different sites and origins might be the potential explanation that miR-155 works in various ways in T cells. According to the bioinformatic prediction, Ptpn2 mRNA was one of the potential binding targets of miR-155, which was reported to induce Treg phenotype transformation and inhibit Th1 and Th17 [28]. In line with the phenotypic alteration of Treg and Th17 upon LPS and hypoxia challenge in response to MSCs, the present study showed Treg phenotypic gene expression of Ptpn2 and Foxp3 mRNA was significantly reduced while Rorc mRNA expression of Th17 phenotypic transcription factor was upregulated in CD4^+^ T cells with miR-155 mimic pretransfection when compared to those without. These findings highlighted that MSC-secreted TGF-*β*1 regulated Treg/Th17 skewing in hypoxic- and LPS-stimulated conditions via miR-155 suppression of CD4^+^ T cells.

Limitations should be noted in our study. First, the study was performed in vitro. Though studies have shown that LPS could activate T cells directly, in vivo environment T cells work with other immune cells rather than work alone. This is why we induced RAW264.7 cells as part of the coculture system and anti-CD3/CD28 antibodies precoated under hypoxic- and LPS-stimulated conditions to mimic the physiological and pathological activation of T cells. Rather than the macrophages that originated from a specific organ, RAW 264.7 is a murine leukemia cell line, which does not reflect activation of immune response linked to an in vivo environment. Though macrophage RAW264.7 cells in coculture settings with CD4^+^ T cells for its expression of CD80, CD86, CD206, and antigen presentation molecules (MHC I/MHCII) especially upon LPS stimulation have been proved [15, 45, 46], the limitation along with it could not be ignored. Second, the coculture of MSCs and CD4^+^ T cells was set at 1 : 5 cell ratio in the present study, which relatively belongs to the high cell ratio coculture [47]. In vitro evidence showed that the effects of MSCs on the phenotypic generation of CD4^+^ T cells are affected by both cell ratio and inflammatory priming [47]. The approach to figure out the effective percentage of injected MSCs in vivo on targeted immune cells remains unsolved with regard to clinical applications. Third, 5% O_2_ was set in vitro for its convenience to reach and commonly used as hypoxia challenge, to investigate how it would impact Treg and Th17 skewing with MSCs. Whether the results differ with varied oxygen concentrations remains to be explored. Fourth, MSCs have been reported to exert exosomes or microvesicles [48], which are able to alleviate inflammation and elicit a therapeutic effect. Whether these could work as the origin of soluble TGF-*β*1 is worthy to be confirmed in further study. Last but not least, MSCs' capacity of multipotential differentiation brings concerns when it comes to clinical application. In the preclinical and clinical studies, MSCs have shown part of benefit on lung protection by attenuating inflammation and reversing progression of fibrosis. In our in vitro coculture system, effects of MSCs on regulating Treg/Th17 skew were not dependent on cell-to-cell contact. For lack of various cell types, the risk related to fibrosis could not be tested in the present study. We have proved that MSCs overexpressing TGF-*β*1 could regulate lung inflammation and attenuate lung injuries by modulating the imbalance of Th17/Treg in the lungs of ARDS mice. The genetic modification would not impact multipotential differentiation ability of MSCs and not increase levels of fibrosis indicators [16]. The risk related to fibrosis remains to be further studied.

## 5. Conclusions

In summary, the study demonstrated that soluble TGF-*β*1 from MSCs restored skewed Treg/Th17 induced by hypoxic- and LPS-stimulated conditions and dampened the inflammation. Additionally, miR-155 suppression of CD4^+^ T cells mediated the regulatory effect of MSC-secreted TGF-*β*1 on skewed Treg/Th17 upon LPS and hypoxia challenge. These findings may provide evidence of MSC application for future T lymphocyte-targeted cell therapy in a specific microenvironment.

## Data Availability

All data generated or analysed during this study are included in this published article.

## Abbreviations

Ab: Antibody
ELISA: Enzyme-linked immunosorbent assay
FCM: Flow cytometry
FoxP3: Forkhead box P3
HGF: Hepatocyte growth factor
LPS: Lipopolysaccharide
miR: MicroRNA
MSCs: Mesenchymal stem cells
PGE2: Prostaglandin E2
ROR*γ*t: Retinoic-acid-receptor-related orphan receptor *γ*t
SLE: Systemic lupus erythematosus
TGF-*β*: Transforming growth factor-beta
Th17: T helper 17
Treg: Regulatory T cell
VEGF: Vascular endothelial growth factor.

## Supplementary Materials

Supplementary Materials Figure S1: the purity of isolated CD4^+^ T cells from mouse spleen using CD4 (L3T4) microbeads was identified by flow cytometric analysis of CD4 staining. Figure S2: the transfection efficiency of miR-155 mimic and control was detected at 4 (A) and 48 (B) hours by qRT-PCR. ^∗^*P* < 0.05 and ^∗∗^*P* < 0.01. Figure S3: Ptpn2 mRNA might be one of the potential targets with 3′-UTR area binding miR-155. Agarwal et al. TargetScanHuman, 2015 (http://www.targetscan.org). Figure S4: graphical abstract. miR-155 suppression of CD4^+^ T cells mediated MSC-secreted TGF-*β*1 modulating skewed Treg/Th17 induced by LPS-hypoxia challenge. Table S1: primer sequence for gene detection.
