# Supplementary material for: Mesenchymal Stem Cell-Secreted TGF-β1 Restores Treg/Th17 Skewing Induced by Lipopolysaccharide and Hypoxia Challenge via miR-155 Suppression
Source: Stem Cells Int. 2022 Mar 12;2022:5522828. doi: 10.1155/2022/5522828 (PMC8934213; doi:10.1155/2022/5522828)
Supplement: Supplementary Materials — Figure S1: the purity of isolated CD4+ T cells from mouse spleen using CD4 (L3T4) microbeads was identified by flow cytometric analysis of CD4 staining. Figure S2: the transfection efficiency of miR-155 mimic and control was detected at 4 (A) and 48 (B) hours by qRT-PCR. ∗P < 0.05 and ∗∗P < 0.01. Figure S3: Ptpn2 mRNA might be one of the potential targets with 3′-UTR area binding miR-155. Agarwal et al. TargetScanHuman, 2015 (http://www.targetscan.org). Figure S4: graphical abstract. miR-155 suppression of CD4+ T cells mediated MSC-secreted TGF-β1 modulating skewed Treg/Th17 induced by LPS-hypoxia challenge. Table S1: primer sequence for gene detection. [file 5522828.f1.docx]

Supplemental Figure S1

Figure
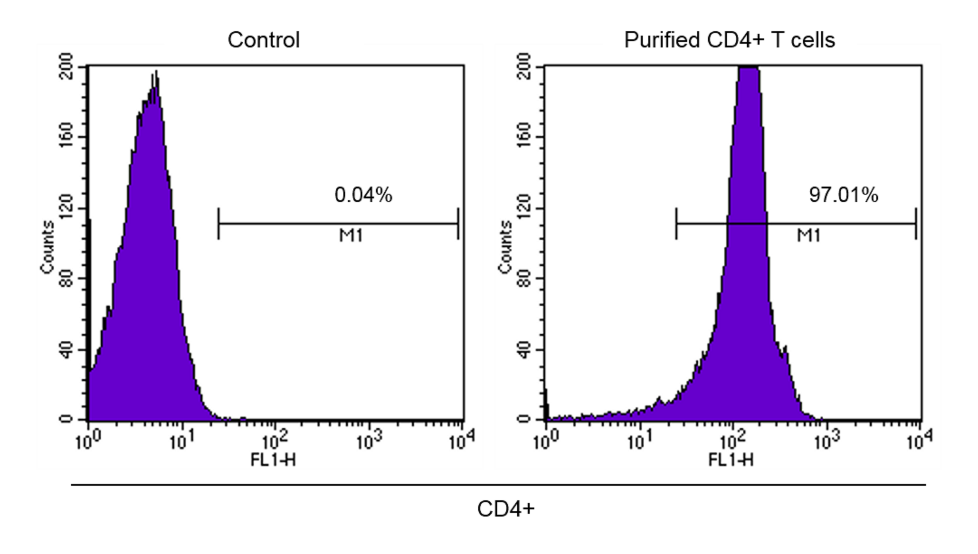
 S1. The purity of isolated CD4^+^T cells from mice spleen using CD4 (L3T4) microbeads was identified by flow cytometric analysis of CD4 staining.

Supplemental Figure S2


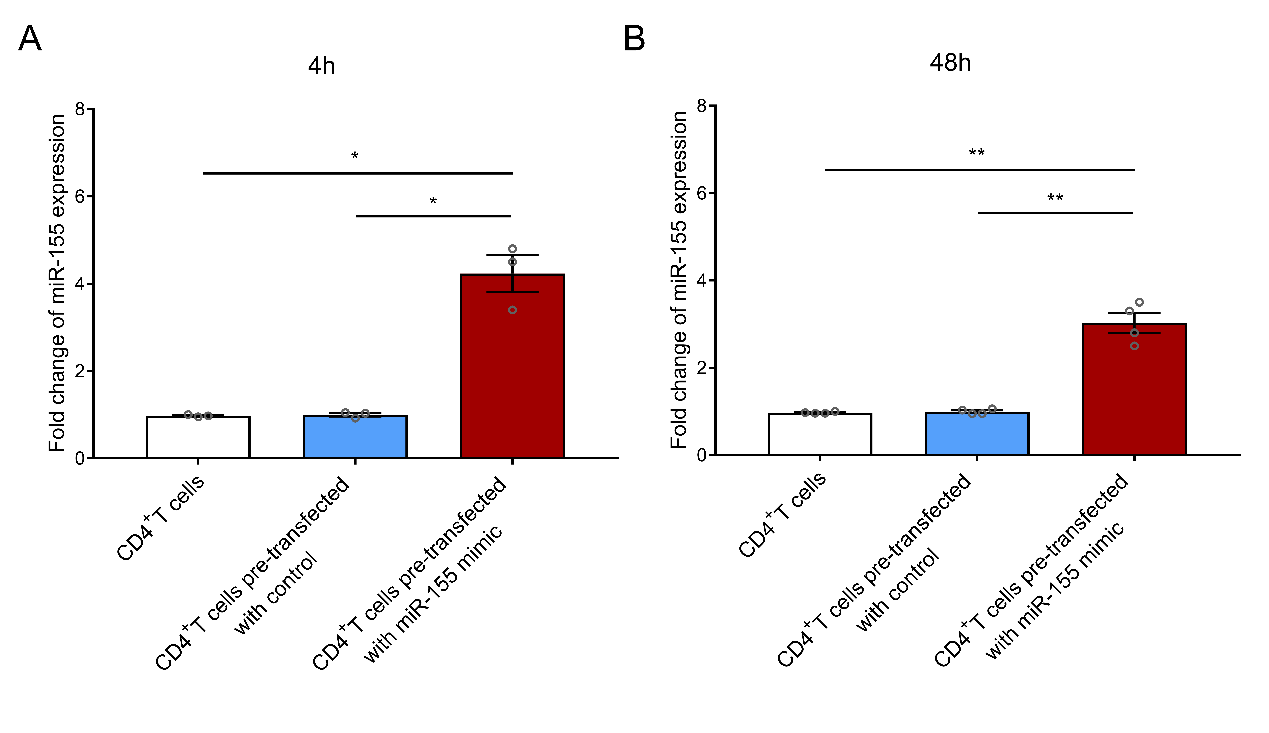


Figure S2. The transfection efficiency of miR-155 mimic and control were detected at 4(A) and 48(B) hours by qRT-PCR. *P<0.05, **P<0.01

Supplemental Figure S
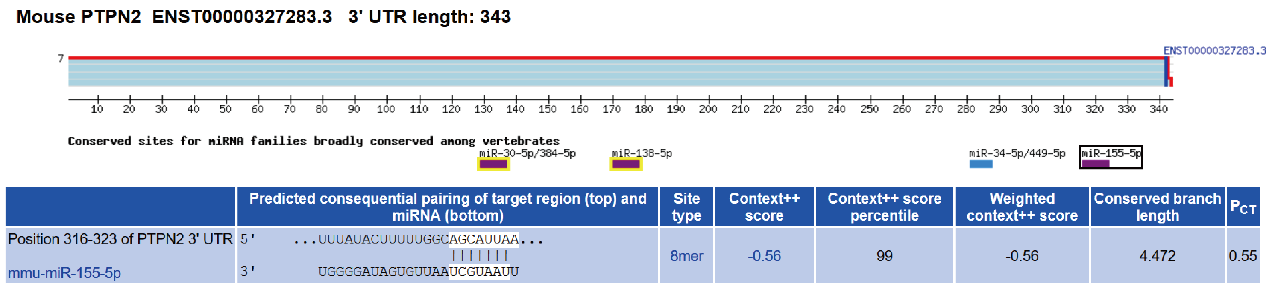
3

Figure S3. Ptpn2mRNA might be one of potential target with 3’-UTR area binding miR-155.

Agarwal et al. TargetScanHuman, 2015. <http://www.targetscan.org>

Supplemental Figure S4


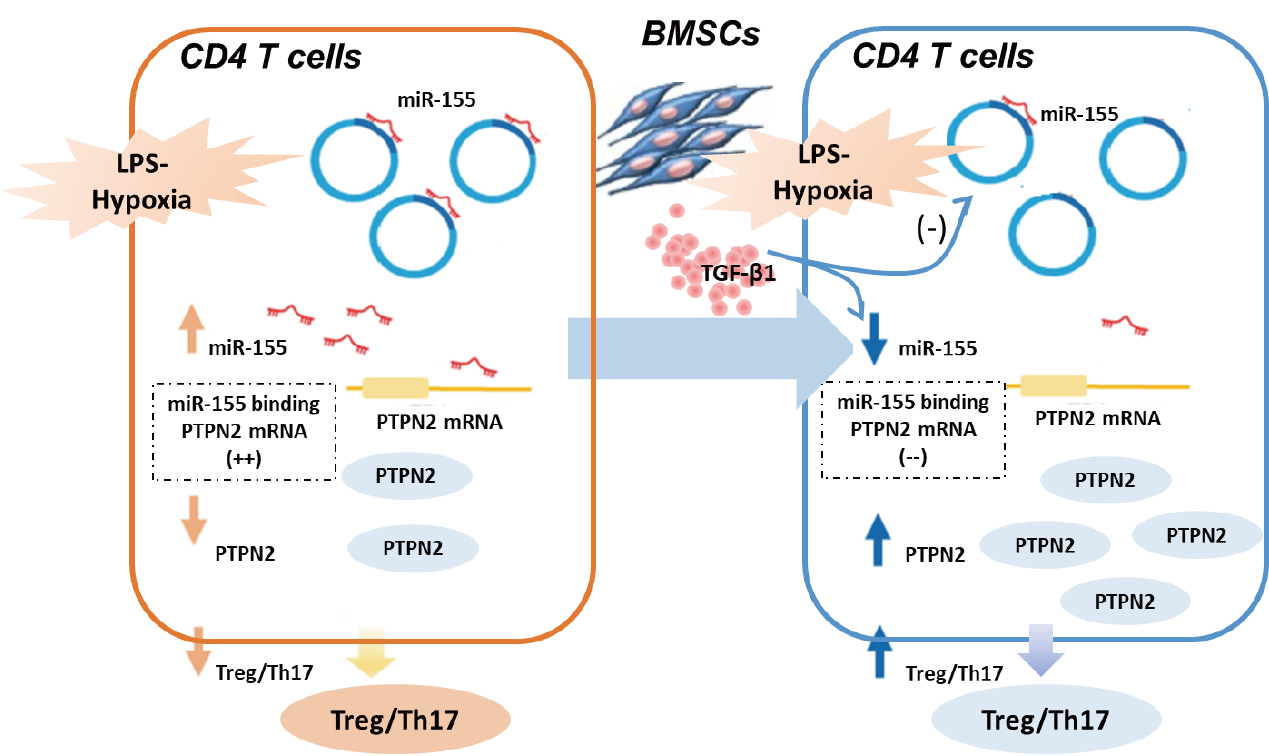


Figure S4. Graphical abstract. MiR-155 suppression of CD4^+^T cells mediated MSCs-secreted TGF-β1 modulating the skewed Treg/Th17 induced by LPS-hypoxia challenge.

Table S1 Primer sequence for gene detection

| Gene | Primer sequence | PCR amplified  Products(bp) |
| --- | --- | --- |
| β-actin | F:5' GTACCACCATGTACCCAGGC 3'  R:5' AACGCAGCTCAGTAACAGTCC 3' | 247 |
| Tgfb1 | F:5' CTTCAGCCTCCACAGAGAAGAACT 3'  R:5' AACGCAGCTCAGTAACAGTCC 3' | 157 |
| Foxp3 | F:5' CTCTAGCAGTCCACTTCACCAA 3'  R:5' CACCCACCCTCAATACCTCTCT 3' | 101 |
| Rorc | F:5' CTGTTTTATGGGGTTTGGGTATGA 3'  R:5' CACCTGTGTGGATGTGTGTCTCTG 3' | 126 |
| Ptpn2 | F:5' CACCTGTGTGGATGTGTGTCTCTG 3'  R:5' TCTTCTGTCATCTGCCCTTCA 3' | 186 |
| U6 | F:5’GCTTCGGCAGCACATATACTAAAAT3’  R:5’CGCTTCACGAATTTGCGTGTCAT3’ | 89 |
| miR-155-5p | GSP:5’GGGGGTTAATGCTAATTGTGAT3’  R:5'GTGCGTGTCGTGGAGTCG3' | 66 |
| miR-146a | GSP:5’GGGTGAGAACTGAATTCC3’  R:5'TGCGTGTCGTGGAGTC3’ | 62 |
